# Buffalo Medical College

**Published:** 1849-04

**Authors:** 


### EDITORIAL DEPARTMENT.

*Buffalo Medical College. Changes in Lecture Term.*—The Faculty of this Institution have determined on some changes as respects the lecture term for the next session. The regular term for 1849–50, will commence on the *first Wednesday in November, and continue sixteen weeks.* There will be a preliminary term of four weeks, attendance being gratuitous and voluntary. During this preliminary term, in addition to dissections, (to which it has hitherto been limited,) clinical instruction will be given in connection with attendance at the hospital, and also daily lectures. It is not intended by the latter, to compromise the instruction belonging to the regular term, but to embrace special subjects which may advantageously be treated more fully than is usually practicable in comprehensive, didactic courses.

The reader will observe that the extension of the regular lecture term, which was promptly adopted by this college in conformity with the recommendation of the National Medical Association, is, for the present, abandoned. Justice to the interests of the college, seems to require this policy, so long as other, and especially proximate institutions, continue to adhere to a four month term. The Buffalo school was the fourth in the order of time, to make the change proposed by the Association. The aggregate of schools that have adopted it, will probably not exceed double the number, and we do not, as yet, observe intimations that their example will be generally followed. We assume to say, as regards this, and any other proposed improvement, that the school with which we are connected, will not be backward in practical conformity, provided its interests will not be materially jeopardized in consequence of the non-concurrence of other institutions, and particularly of those institutions with whom it must stand somewhat in the relation of competition. But, until then, in so far as this measure is concerned, the friends of the institution must be content with the disposition which has been evinced by the trial which has been made,and by endeavoring to secure the advantages of a lengthened period of instruction, by means of lectures, etc., during a preliminary term which students are invited to attend gratuitously, but which is not obligatory, and will not interfere with the regular term.

Those of our readers who may recollect the tenor of former editorial remarks on this topic, are aware, that we have, from the first, advocated the propriety of an extension of the term of lectures in medical colleges. We have done so under the belief that the various branches of medical education are too extensive to be fully and satisfactorily taught in so short a period. We now write, after an opportunity of having tested somewhat the practical working of the plan of continuing lectures for a longer period, and we are free to say, that we are less disposed, than heretofore, to urge its importance. We are now nearly at the close of, virtually, a six months' session, and we can realize, what before we had imperfectly appreciated, that the unremitted continuance of labors for so long a period becomes fatiguing, for both teacher and pupil, to a degree calculated to compromise their usefulness. While, therefore, it must be admitted, that a four months' term, divided among the several departments of medical instruction, is too short, in proportion to the amount of information to be communicated, we are not satisfied that an extension of the time to six months, is the best method of improvement. We are not prepared, at present, to submit a plan to be preferred, but would leave the subject to be discussed by others, and perhaps, also, by us at a future time.

The new edifice, now in process of completion, will be in readiness for occupancy at the commencement of the next session of the Buffalo Medical College.

